# Journal title change

**DOI:** 10.5713/ab.2021.0001ED

**Published:** 2020-12-22

**Authors:** Jong K. Ha

**Affiliations:** 1 Animal Bioscience, Seoul 08776, Korea; 2 Department of Agricultural Biotechnology, College of Agriculture and Life Science, Seoul National University, Seoul 08826, Korea

The *Asian-Australasian Journal of Animal Sciences* (*AJAS*) was established in March 1988 as the official journal of the Asian-Australasian Association of Animal Production Societies (AAAP). The journal’s mission was to serve the animal agricultural industry and academia in the Asian-Australasian region through the efficient publication and distribution of information on animal science. Since its inception, the *AJAS* has made tremendous contributions to the development of animal science in the Asian-Australasian region over the past 30 years. The *AJAS* now publishes more than 200 original articles and review papers over 12 issues per year, which is a substantial leap from 33 articles over four issues in the first few years. A more dramatic increase was observed in the number of submissions, which grew from fewer than 50 to 1,000 annually.

To further increase its international representation, the editorial board has decided to change the title of the journal from the ***Asian-Australasian Journal of Animal Sciences*** (*AJAS*) to ***Animal Bioscience*** (*AB*), effective from January 2021 (Volume 34, No. 1). We hope that this change will enhance the journal’s brand identity by providing a clearer and more distinct message, enabling the journal to draw more international readership, and providing us with a greater opportunity to encompass diverse interests of future animal agricultural science and industry in the AAAP region.

***Animal Bioscience*** will continue to publish high-quality, original research and review articles in the area of animal breeding and genetics, reproduction, nutrition, products, health, welfare, and the environment. We also hope that the new title will contribute to creating and fostering new emerging areas of animal sciences. Considering our past contribution to animal industry and academia through our publications and future direction toward becoming a top archival journal, the new title will attract more attention from authors, subscribers, and readers.

All manuscripts in various processing stages (review, revision, editing, etc.), once accepted, will be published under the new journal title, ***Animal Bioscience***, beginning with the January 2021 issue (Volume 34, No. 1). All submissions from January 2021 must be completed via the new online system (animbiosci.org). You can contact us at animbiosci@gmail.com (effective from January 1, 2021); however, the older email address (jongkha@hotmail.com) will also be functional until the end of 2021. Please do not hesitate to contact us if you have any questions regarding the change of the journal title or other publication-related matters.

